# Supplementary material for: Solvent vapor diffusion–driven multiscale pre-aggregation of non-fullerene acceptors enables high-performance organic solar cells
Source: Nat Commun. 2025 Dec 17;16:11188. doi: 10.1038/s41467-025-66199-5 (PMC12712035; doi:10.1038/s41467-025-66199-5)
Supplement: Supplementary file 1 — Supplementary Information [file 41467_2025_66199_MOESM1_ESM.pdf]

Supplementary Information

Solvent vapor diffusion–driven multiscale pre-aggregation of non-fullerene acceptors enables high-performance organic solar cells

Weilin Zhou,[1] Xingjian Dai,[1] Ben Fan,[1] Hongxiang Li,[2] Xiaopeng Xu,[1,*] Yihui Wu,[1] Qiang Peng[1,*]

[1]School of Chemical Engineering and State Key Laboratory of Advanced Polymer Materials, Sichuan University, Chengdu 610065, P. R. China.

[2]College of Polymer Science and Engineering, Sichuan University, Chengdu 610065, P. R. China.

Corresponding Authors: *To whom correspondence should be addressed: Tel: +86-28-86510868; fax: +86-28-86510868; e-mail: xpxu@scu.edu.cn; qiangpeng@scu.edu.cn

Table of Contents

Supplementary Figures 3

Supplementary Fig. 1. 3

Supplementary Fig. 2. 4

Supplementary Fig. 3. 5

Supplementary Fig. 4. 6

Supplementary Fig. 5. 7

Supplementary Fig. 6. 8

Supplementary Fig. 7. 9

Supplementary Fig. 8. 10

Supplementary Fig. 9. 11

Supplementary Fig. 10. 12

Supplementary Fig. 11. 13

Supplementary Fig. 12. 14

Supplementary Fig. 13. 15

Supplementary Fig. 14. 16

Supplementary Fig. 15. 17

Supplementary Fig. 16. 18

Supplementary Fig. 17. 19

Supplementary Fig. 18. 20

Supplementary Fig. 19. 21

Supplementary Fig. 20. 22

Supplementary Fig. 21. 23

Supplementary Fig. 22. 24

Supplementary Fig. 23. 25

Supplementary Fig. 24. 26

Supplementary Fig. 25. 27

Supplementary Fig. 26. 28

Supplementary Fig. 27 29

Supplementary Fig. 28. 30

Supplementary Fig. 29. 31

Supplementary Fig. 30. 32

Supplementary Fig. 31. 33

Supplementary Fig. 32. 34

Supplementary Fig. 33. 35
Supplementary Tables 36
Supplementary Table 1 36
Supplementary Table 2 37
Supplementary Table 3 38
Supplementary Table 4 39
Supplementary Table 5 40
Supplementary Table 6 41
Supplementary Table 7 42
Supplementary Table 8 43
Supplementary Table 9 44
Supplementary Table 10 45
Supplementary Table 11 46
Supplementary Table 12 47
Supplementary Table 13 48

Supplementary Figures

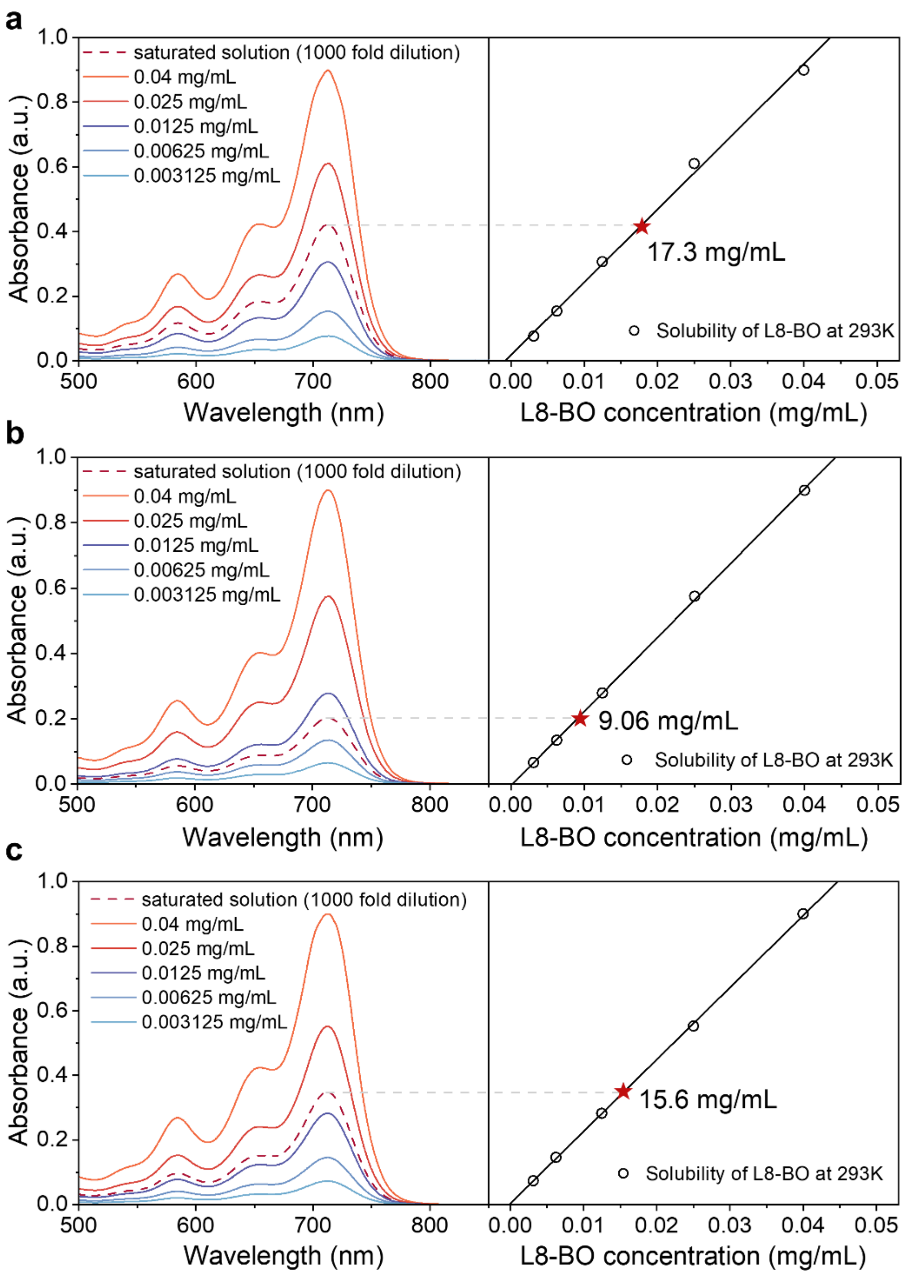

Supplementary Fig. 1. Solubility determination of L8-BO in various solvents by UV–vis absorption. Absorption spectra were recorded for L8-BO solutions in three different solvent conditions at concentrations ranging from 0.003125 to 0.04 mg/mL, as well as for a 1000-fold diluted saturated solution. A linear correlation between absorbance and concentration was observed, enabling the determination of the saturated solubility of L8-BO. The measured saturated concentrations of L8-BO were 17.3 mg/mL in toluene **a**, 9.06 mg/mL in benzene **b**, and 15.6 mg/mL in toluene/benzene mixed solvent according to SVD-20 **c**.

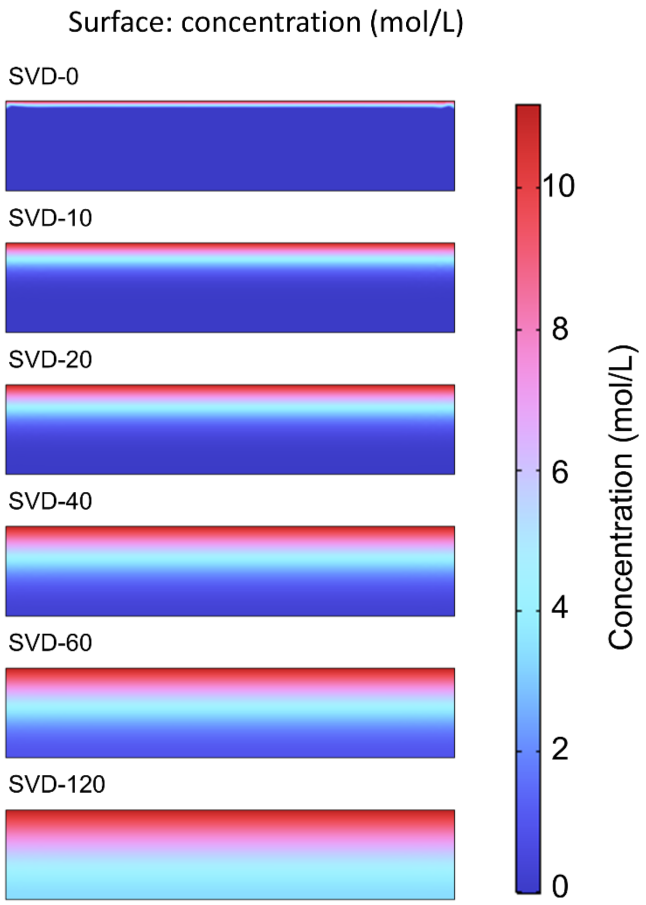

Supplementary Fig. 2. COMSOL simulation showing benzene concentration gradient evolution as a function of SVD time.

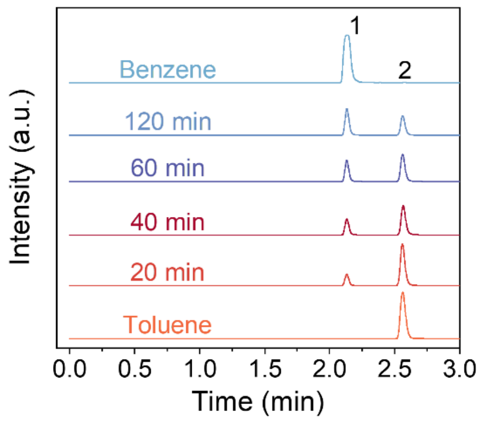

Supplementary Fig. 3. The benzene and toluene fractions in mixed solvent after different SVD time determined gas chromatograms. Peak 1 represents benzene, and peak 2 represents toluene.

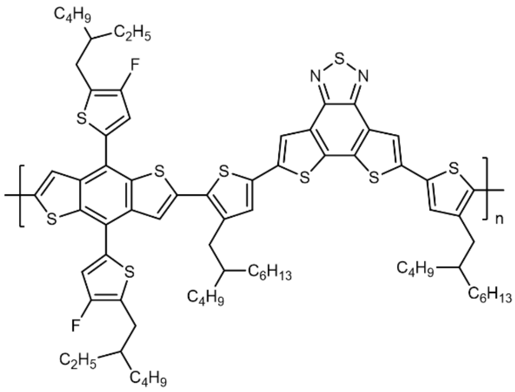

Supplementary Fig. 4. Chemical structure of D18. It is used as the donor material for all devices, unless otherwise specified.

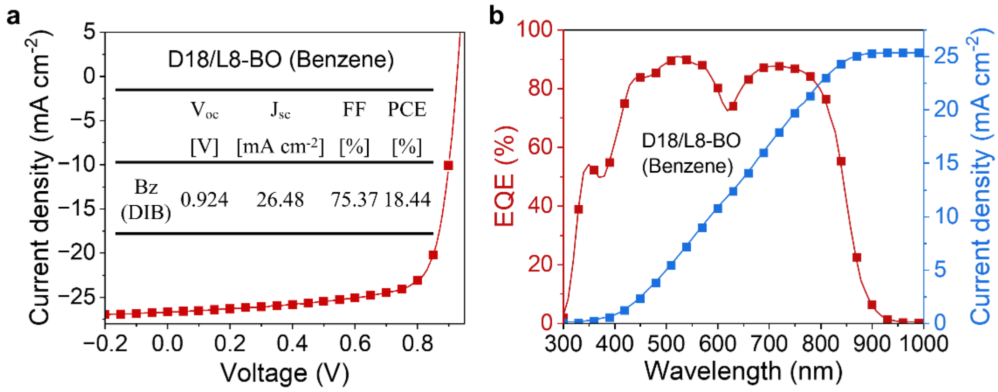

Supplementary Fig. 5. Performance of D18/L8-BO device using benzene (Bz) as the solvent to dissolve L8-BO acceptor and DIB as the additive. **a** *J*-*V* curve. **b** EQE spectrum.

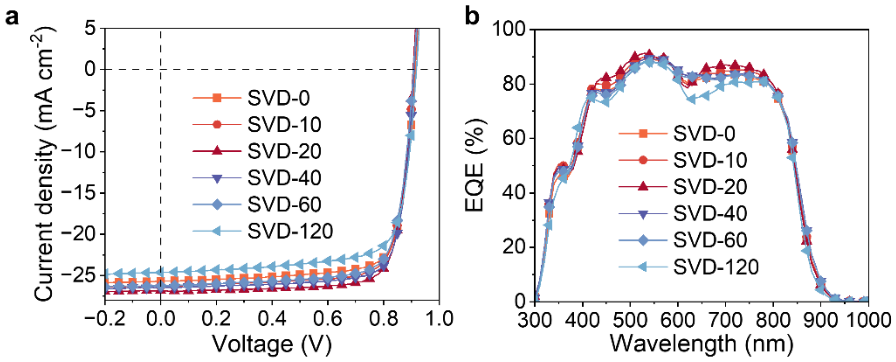

Supplementary Fig. 6. Performance of D18/L8-BO devices processed by varying SVD durations without additive. **a** *J*-*V* curves. **b** EQE spectra.

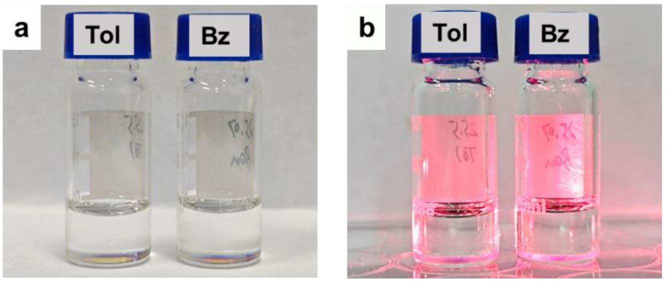

Supplementary Fig. 7. Optical phenomena of DIB at a concentration of 50 mg/mL in benzene (Bz) and toluene (Tol). **a** Without laser irradiation. **b** With laser irradiation.

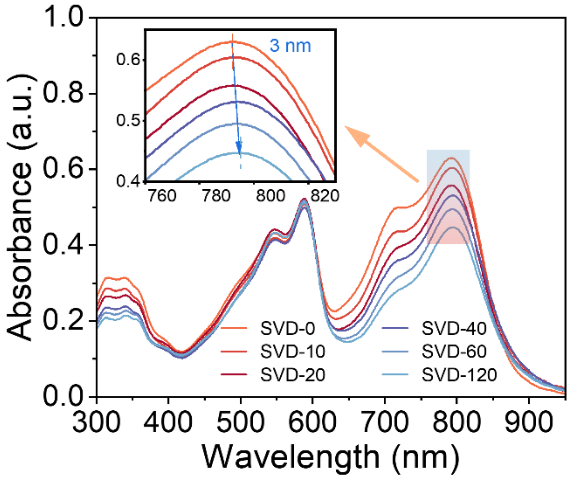

Supplementary Fig. 8. UV–vis–NIR absorption spectra of D18/L8-BO thin films. The films were prepared by depositing L8-BO solutions with various SVD durations, followed by spin-coating onto D18 layers.

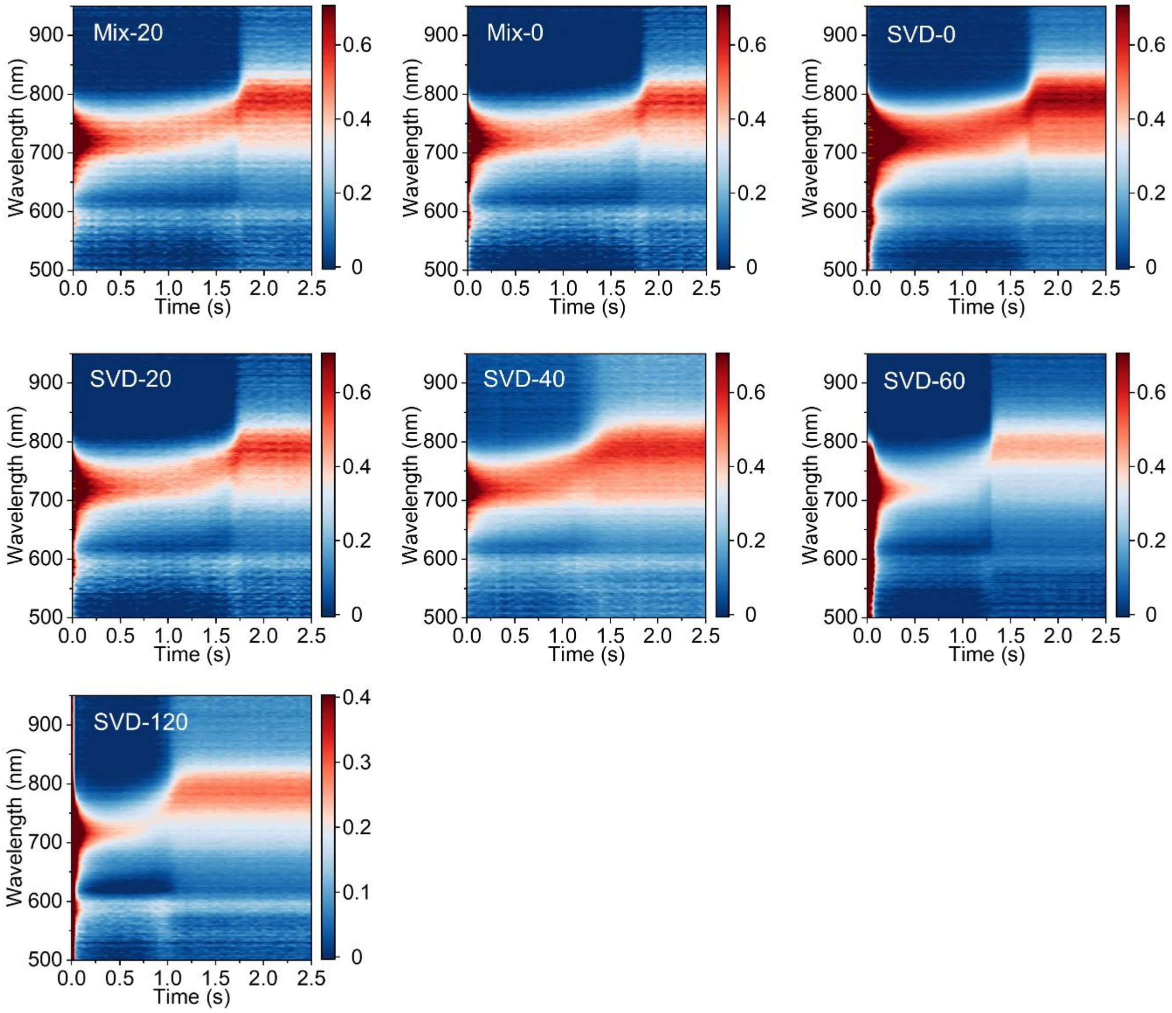

Supplementary Fig. 9. 2D time-resolved in-situ absorption spectra of D18/L8-BO films. These spectra were obtained during the spin-coating of differently treated L8-BO solutions onto D18 layers.

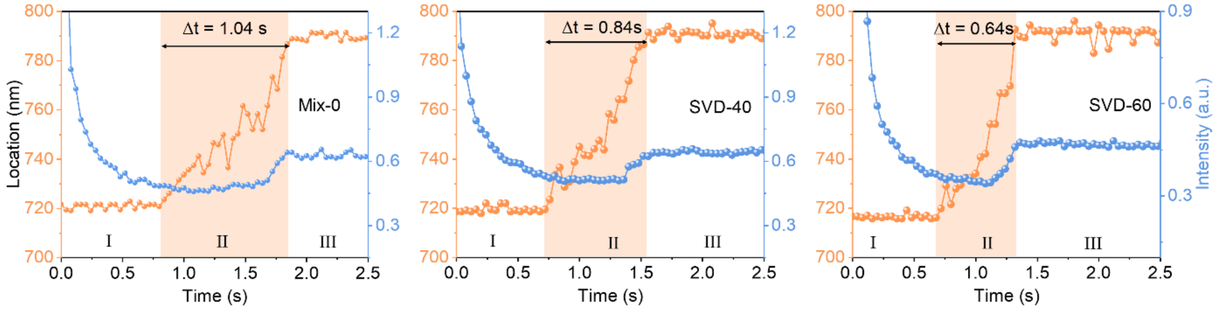

Supplementary Fig. 10. The L8-BO absorption peak location and intensity kinetics extracted from 2D time-resolved in-situ absorption spectra.

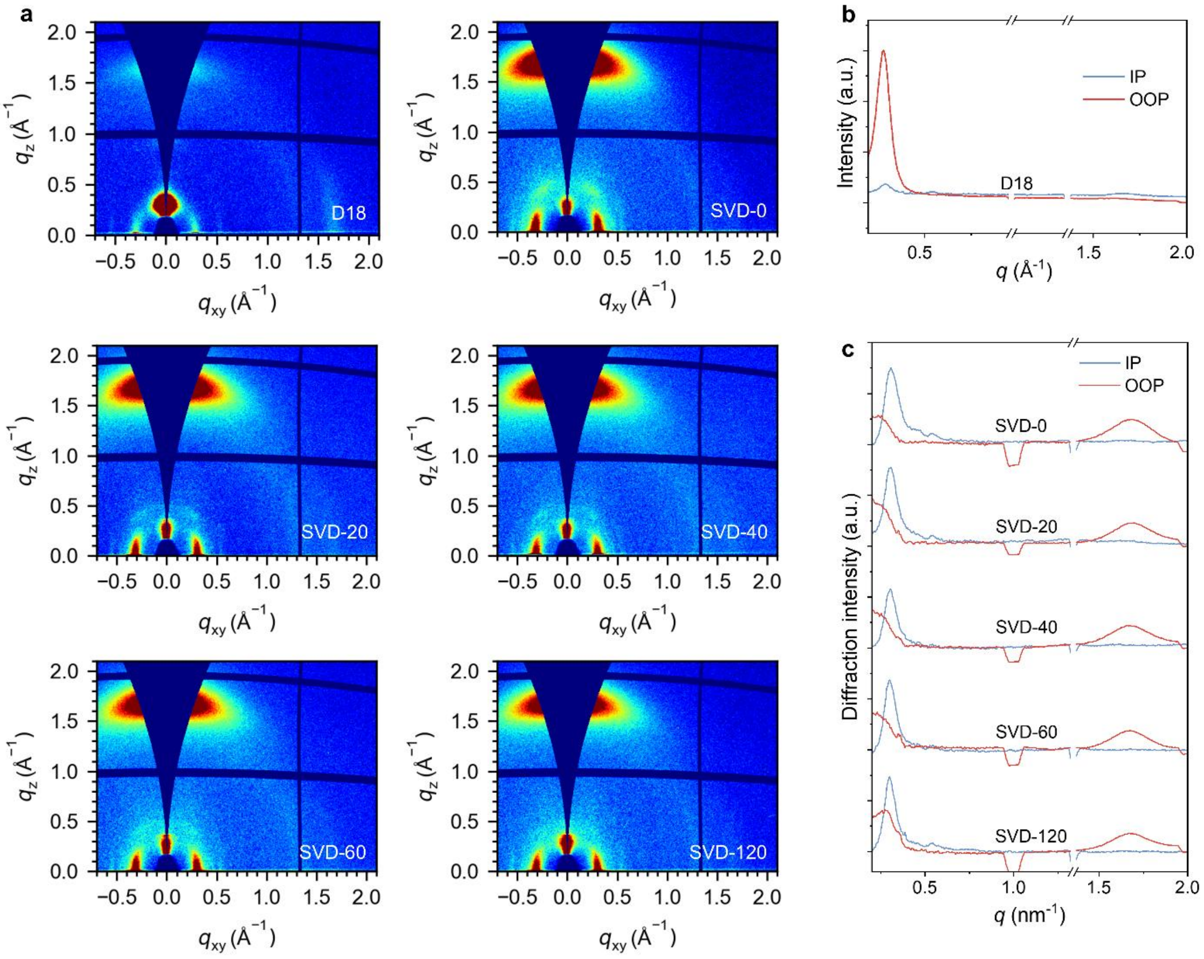

Supplementary Fig. 11. Molecular packing of neat D18 film and D18/L8-BO films processed under different conditions. **a** 2D GIWAXS patterns. **b**,**c** The corresponding IP and OOP line-cuts derived from **a**.

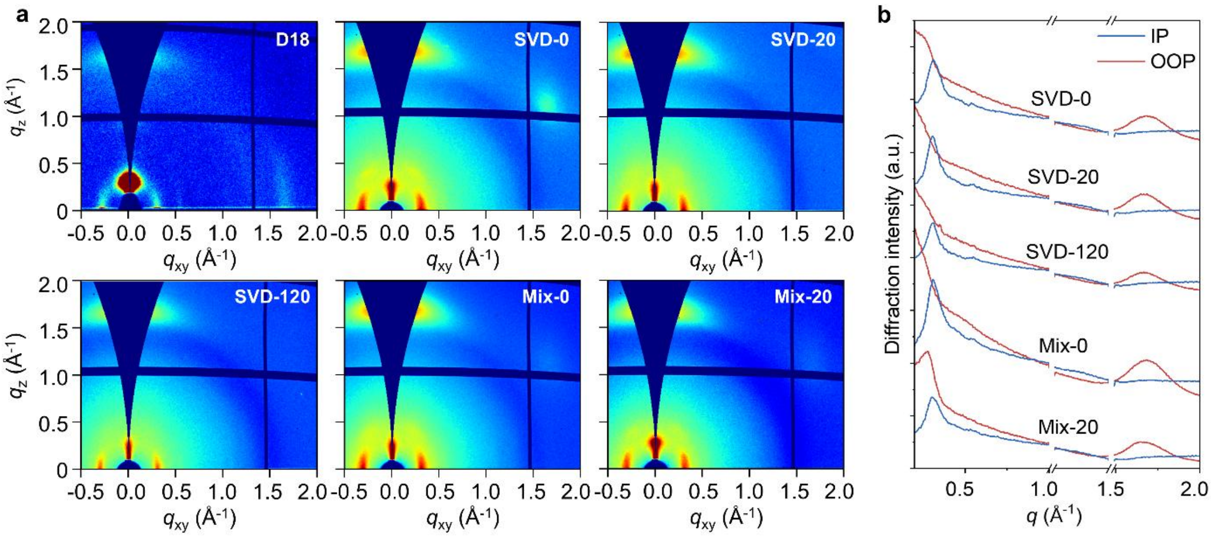

Supplementary Fig. 12. Molecular packing of neat D18 film and D18/L8-BO films processed under different conditions. **a** 2D GIWAXS patterns. **b** The corresponding IP and OOP line-cuts derived from **a**.

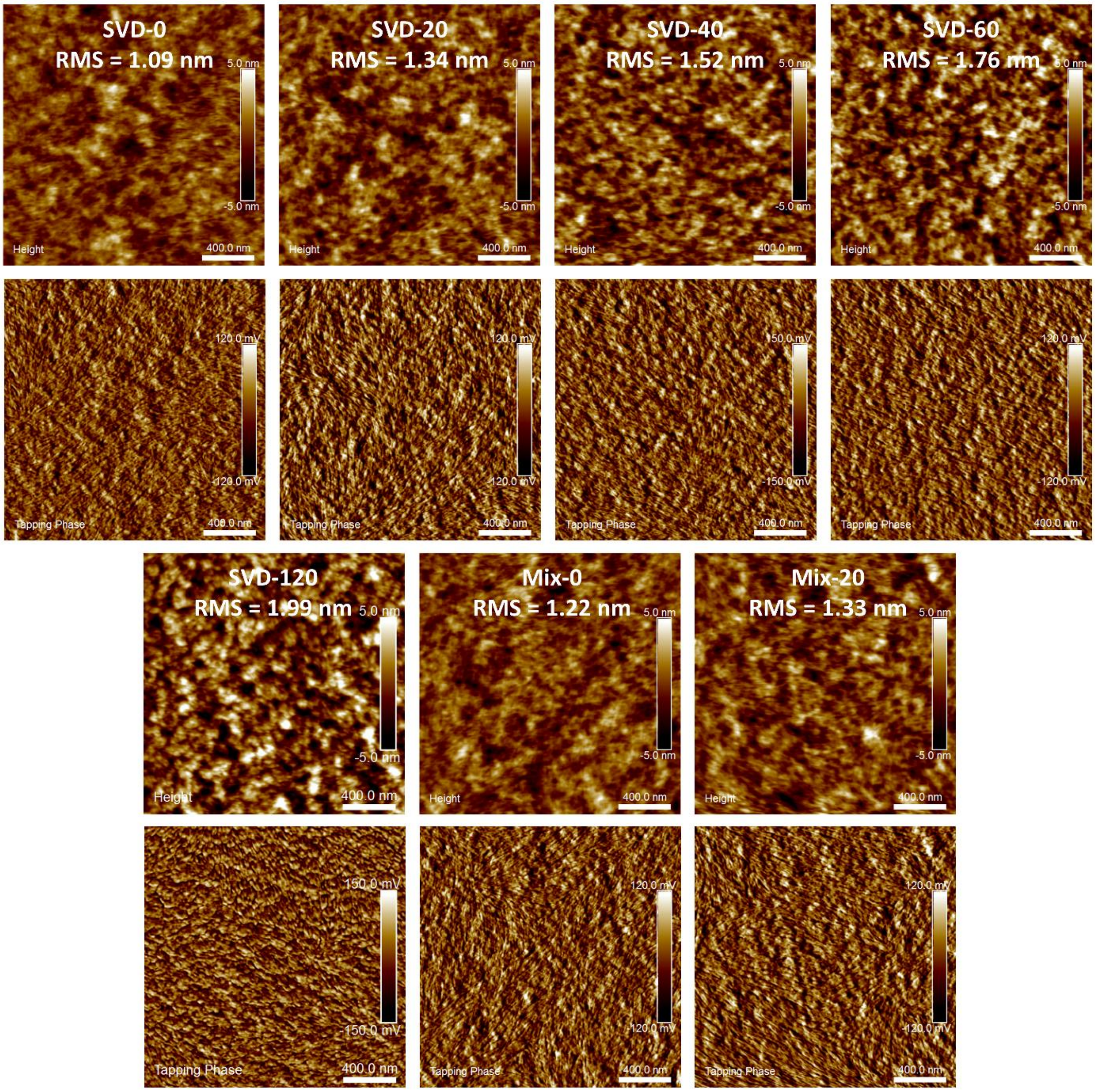

Supplementary Fig. 13. AFM height and tapping phase images of the D18/L8-BO films processed under different conditions.

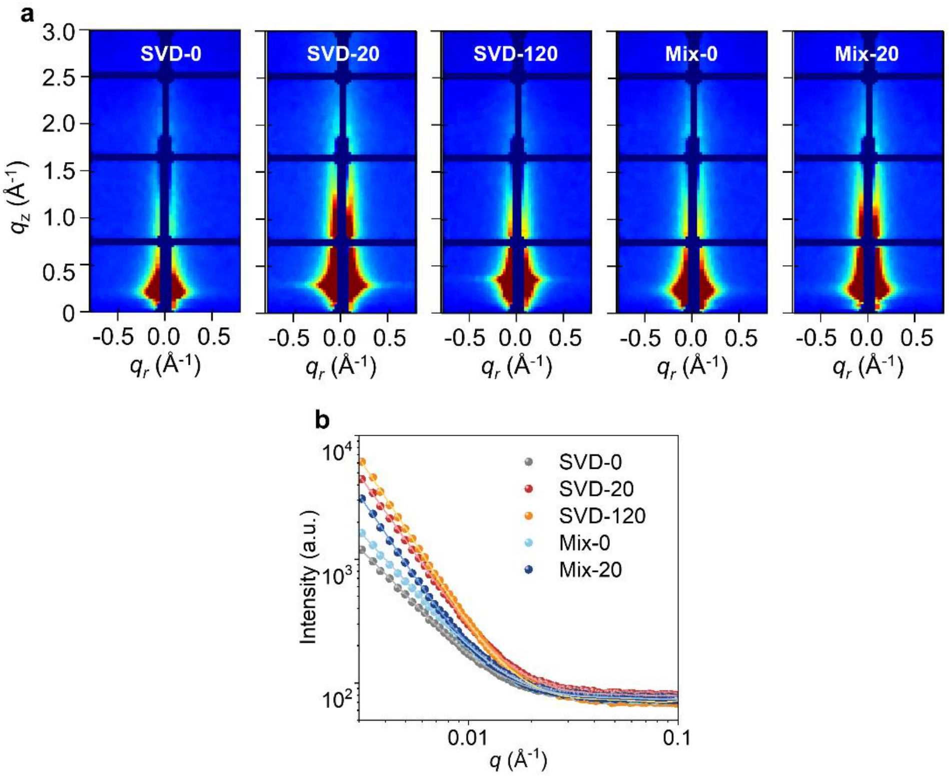

Supplementary Fig. 14. a GISAXS patterns of D18/L8-BO films processed under various conditions. **b** Scattering intensity profiles derived from **a**.

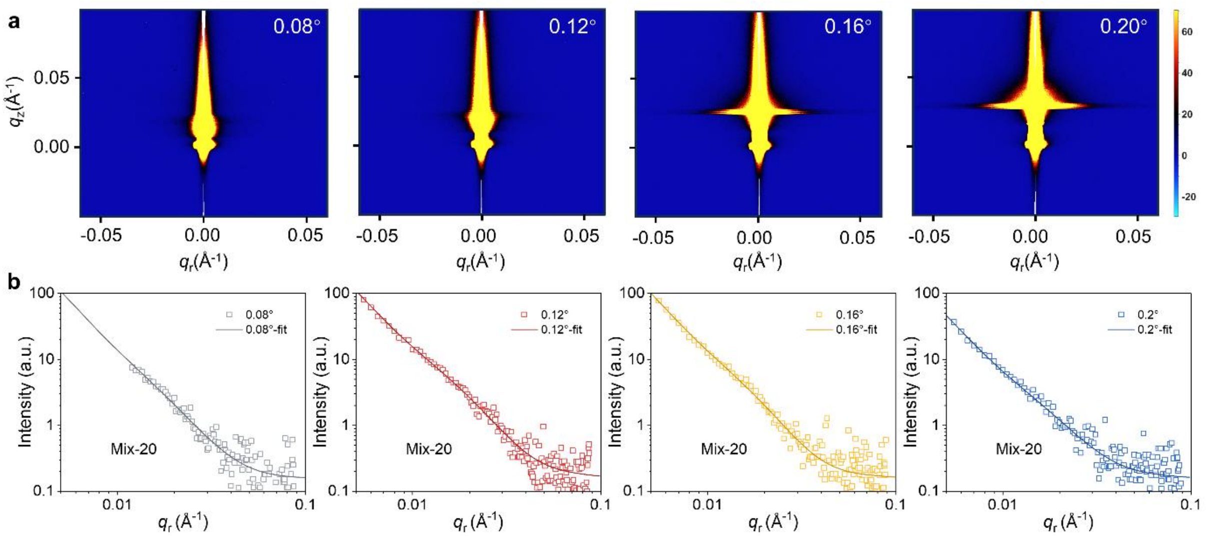

Supplementary Fig. 15. GTSAXS measurements of the Mix-20 film. **a** 2D patterns at varing incident angles. **b** The corresponding q_r line-cut profiles and fitting curves.

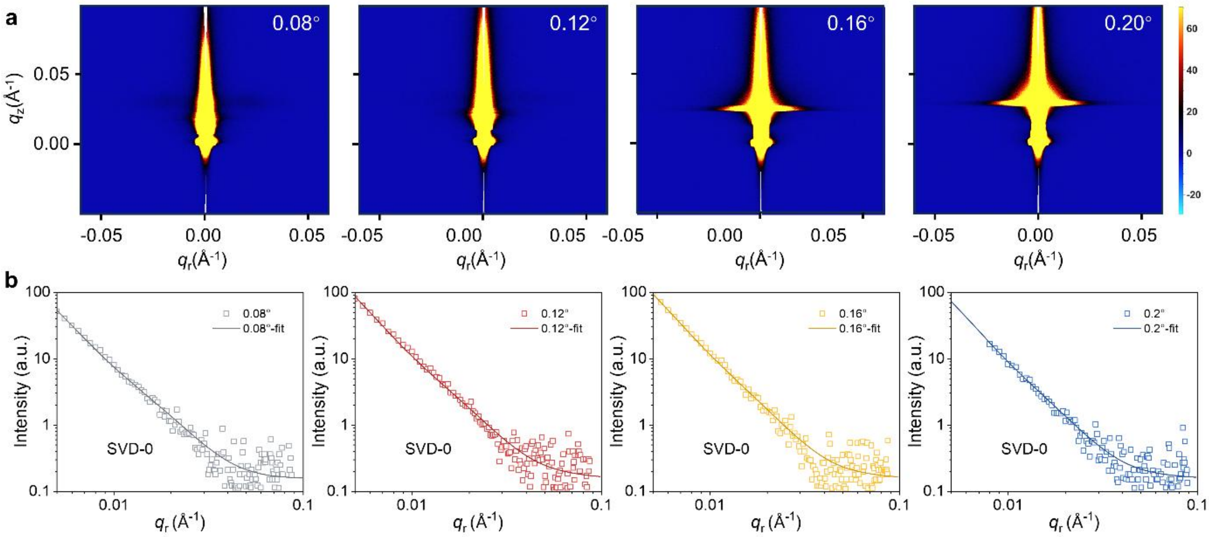

Supplementary Fig. 16. GTSAXS measurements of the SVD-0 film. **a** 2D patterns at varing incident angles. **b** The corresponding q_r line-cut profiles and fitting curves.

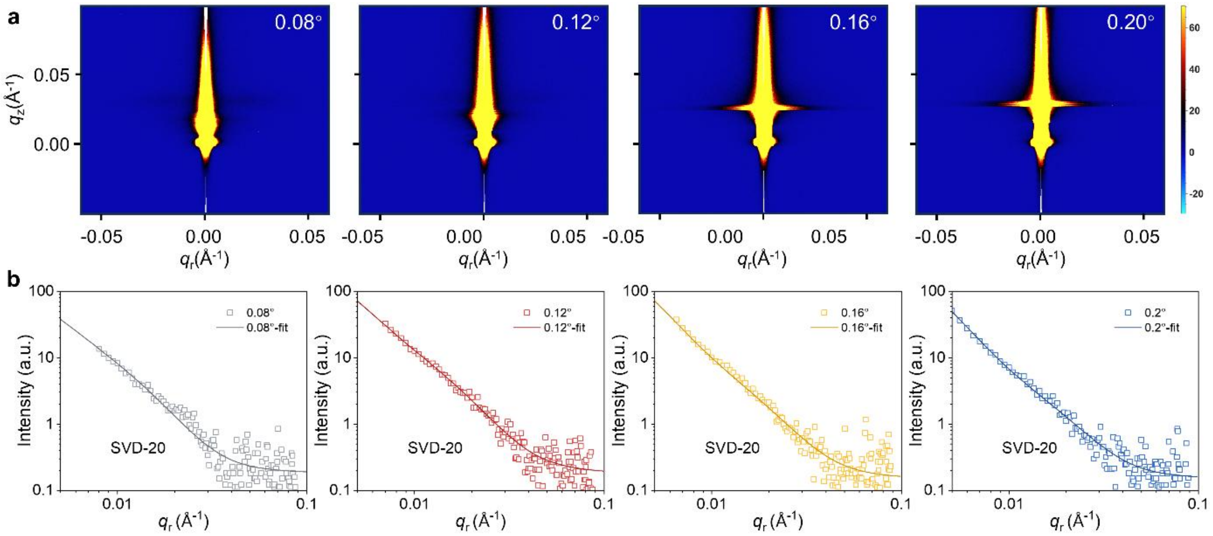

Supplementary Fig. 17. GTSAXS measurements of the SVD-20 film. **a** 2D patterns at varing incident angles. **b** The corresponding q_r line-cut profiles and fitting curves.

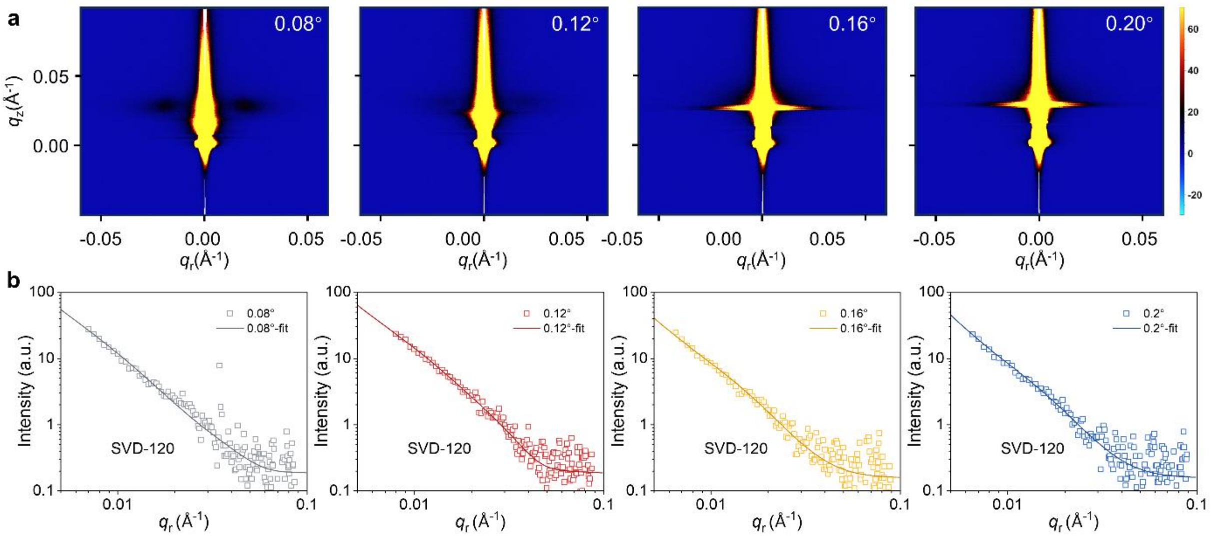

Supplementary Fig. 18. GTSAXS measurements of the SVD-120 film. **a** 2D patterns at varing incident angles. **b** The corresponding q_r line-cut profiles and fitting curves.

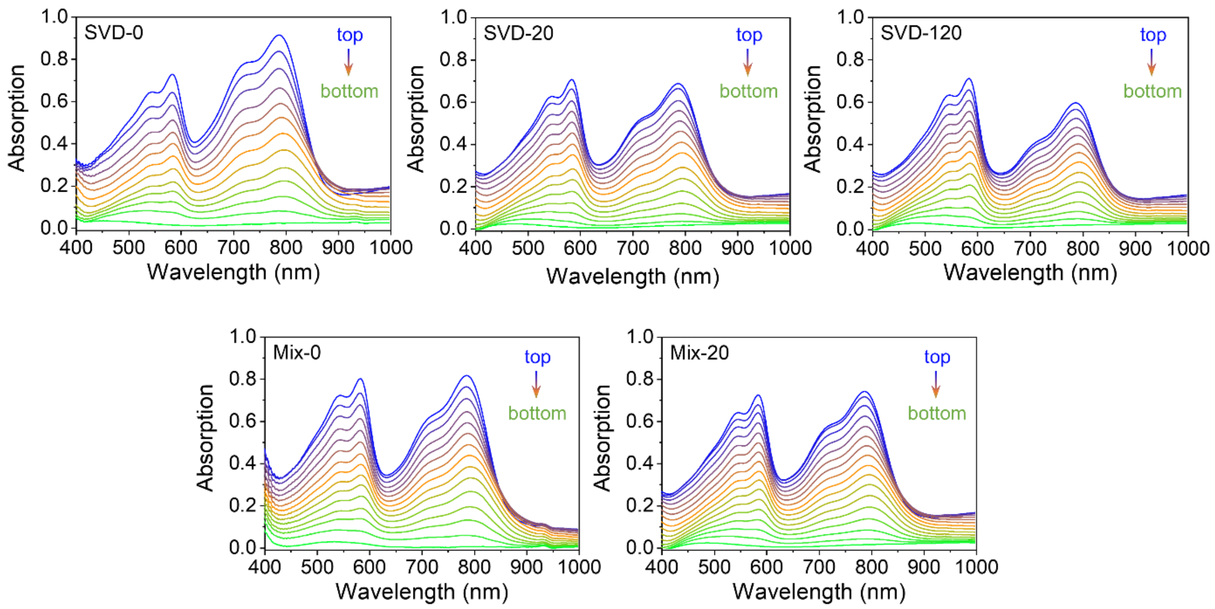

Supplementary Fig. 19. Film-depth-dependent absorption spectroscopy of D18/L8-BO films processed under different conditions.

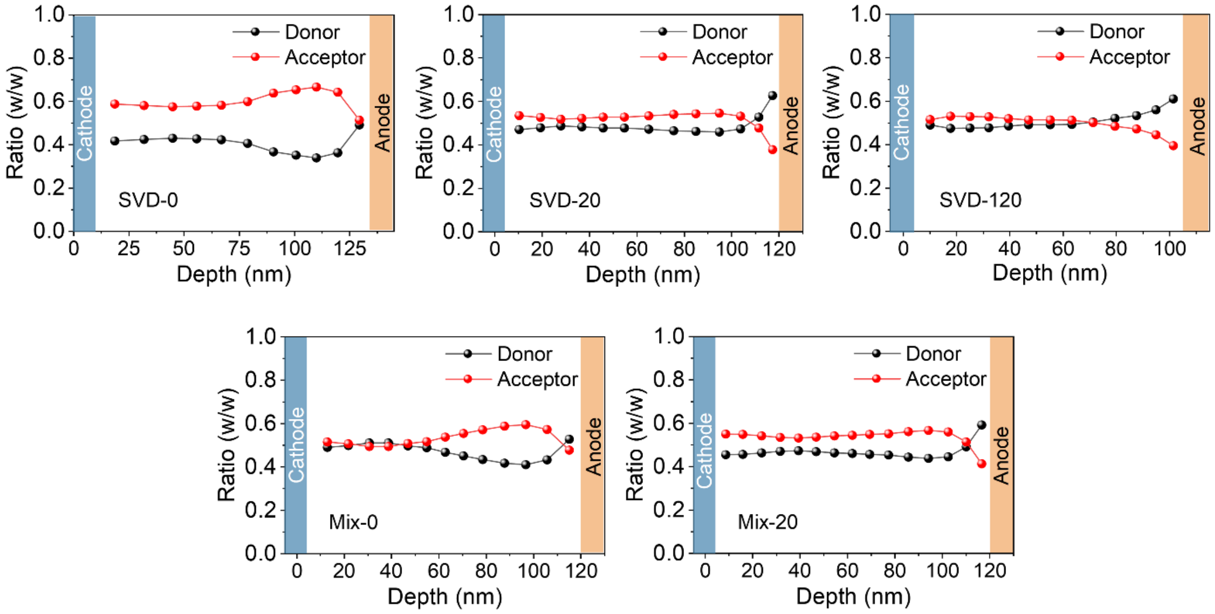

Supplementary Fig. 20. Component distribution profiles at different depths of D18/L8-BO films processed under different conditions.

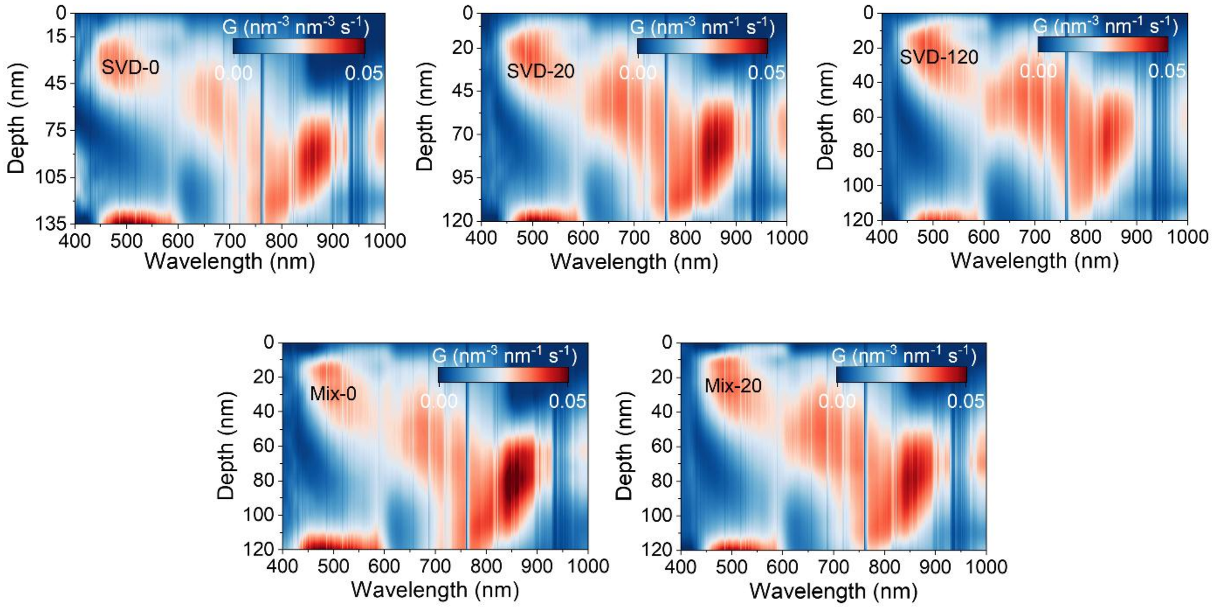

Supplementary Fig. 21. Calculated exciton generation contours under different conditions (unit, 10^{25} nm^{-3} nm^{-1} s^{-1}).

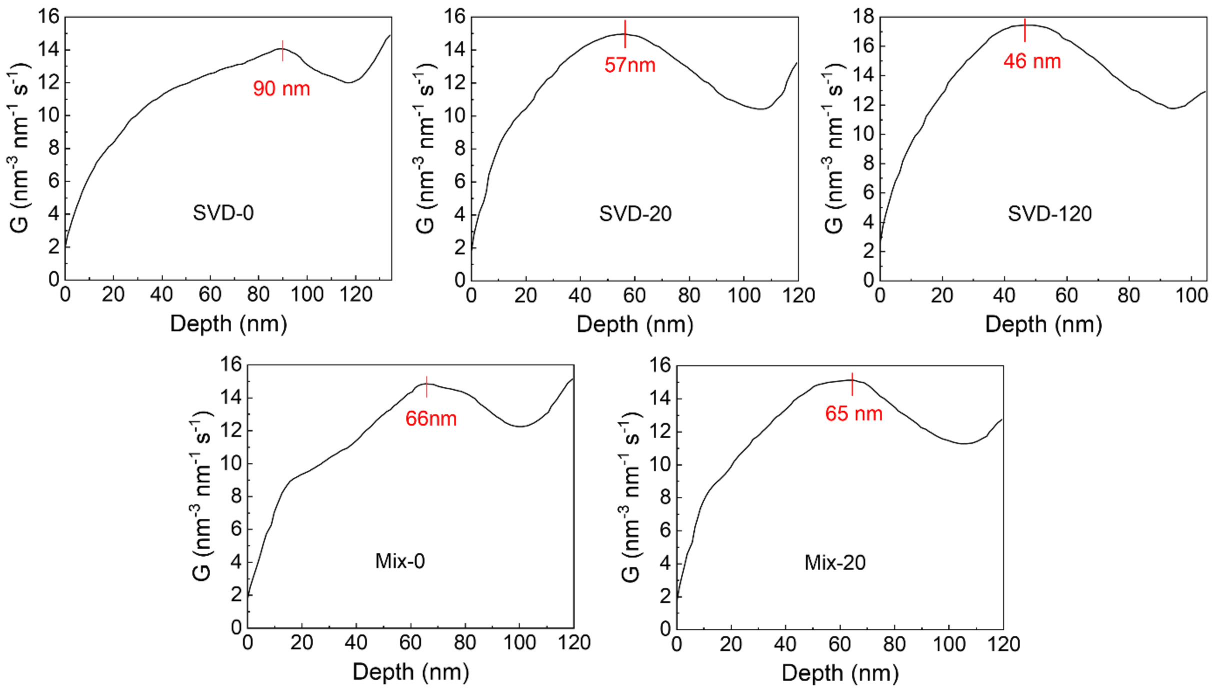

Supplementary Fig. 22. Film-depth-dependent exciton generation rate (G) of the active layers under different conditions. (unit,10^{27} nm^{-3} nm^{-1} s^{-1}).

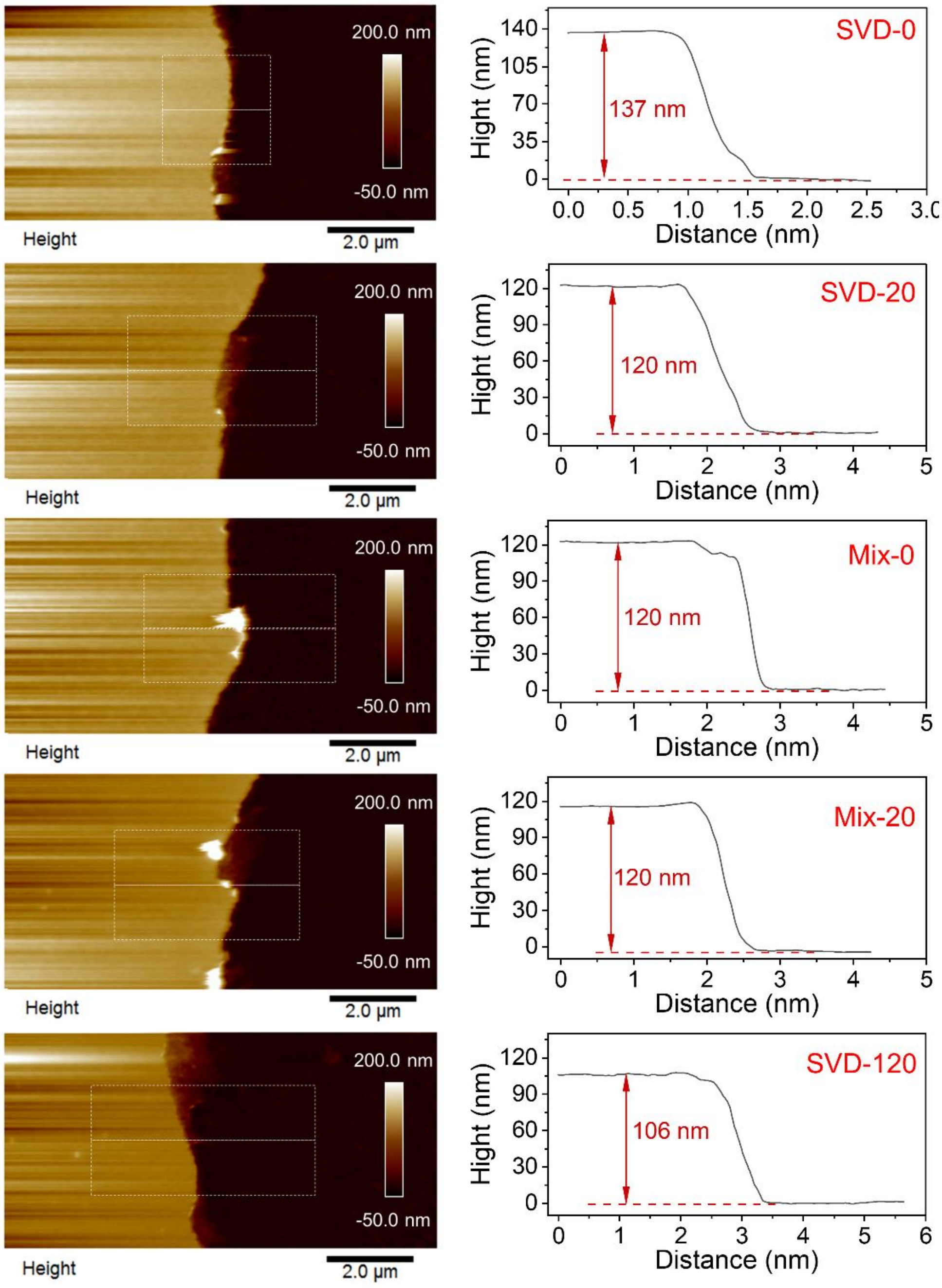

Supplementary Fig. 23. Film thicknesses of the D18/L8-BO films measured by AFM.

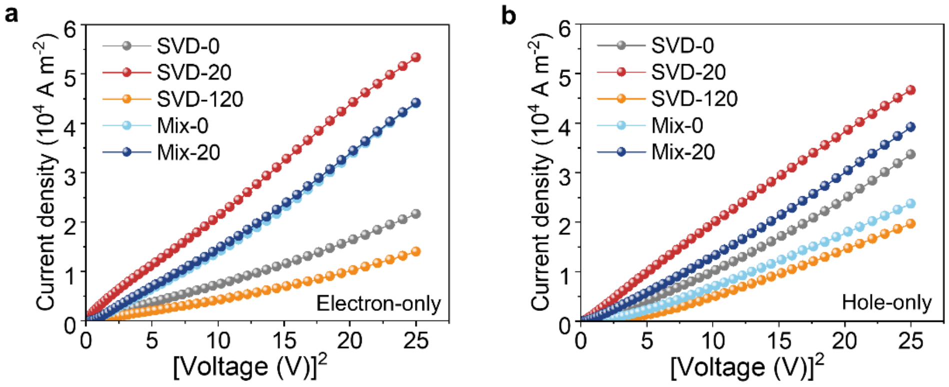

Supplementary Fig. 24. J-V^2 curves of unipolar devices based on D18/L8-BO processed under different conditions. **a** electron-only devices. **b** hole-only devices.

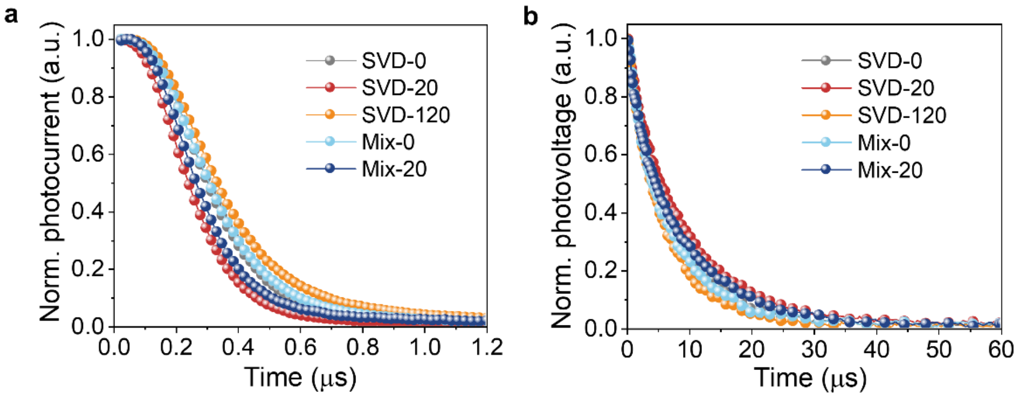

Supplementary Fig. 25. a Normalized transient photocurrent (TPC) of D18/L8-BO based OSCs. **b** Normalized transient photovoltage (TPV) of D18/L8-BO based OSCs.

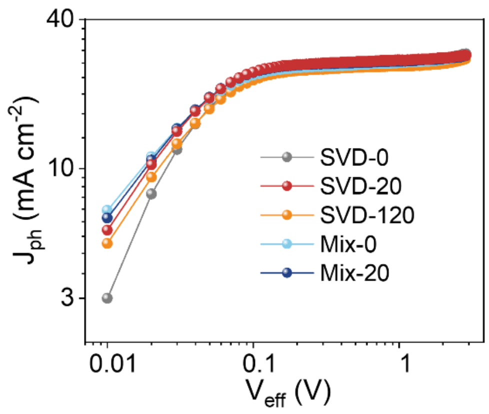

Supplementary Fig. 26. The correlation between photocurrent density (J_{ph}) and effective voltage (V_{eff}) of D18/L8-BO based OSCs.

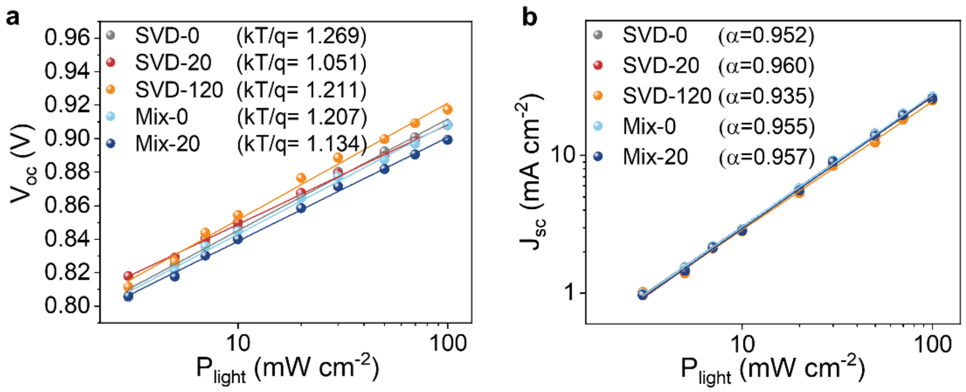

Supplementary Fig. 27. Charge recombination in D18/L8-BO based OSCs. a, V_{OC} versus P_{light} plots. **b**, J_{SC} versus P_{light} plots.

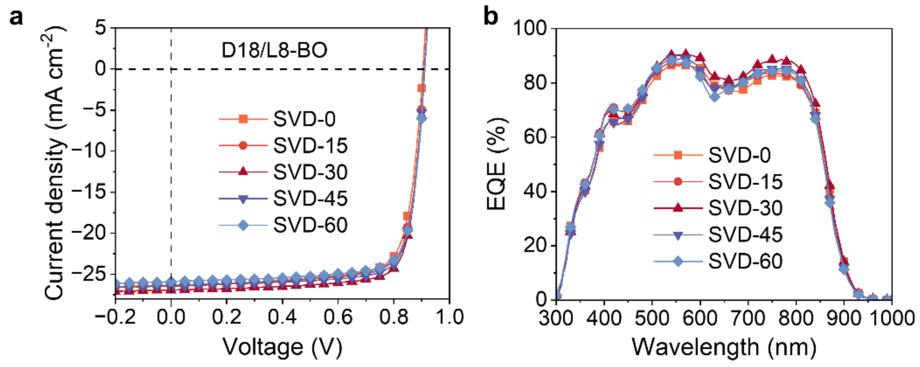

Supplementary Fig. 28. Photovoltaic performances of D18/L8-BO based OSCs. Chloroform was employed as the donor solvent, dioxane/chlorobenzene was employed as solvent pair for SVD treatment of L8-BO acceptor. **a** *J-V* curves. **b** EQE spectra.

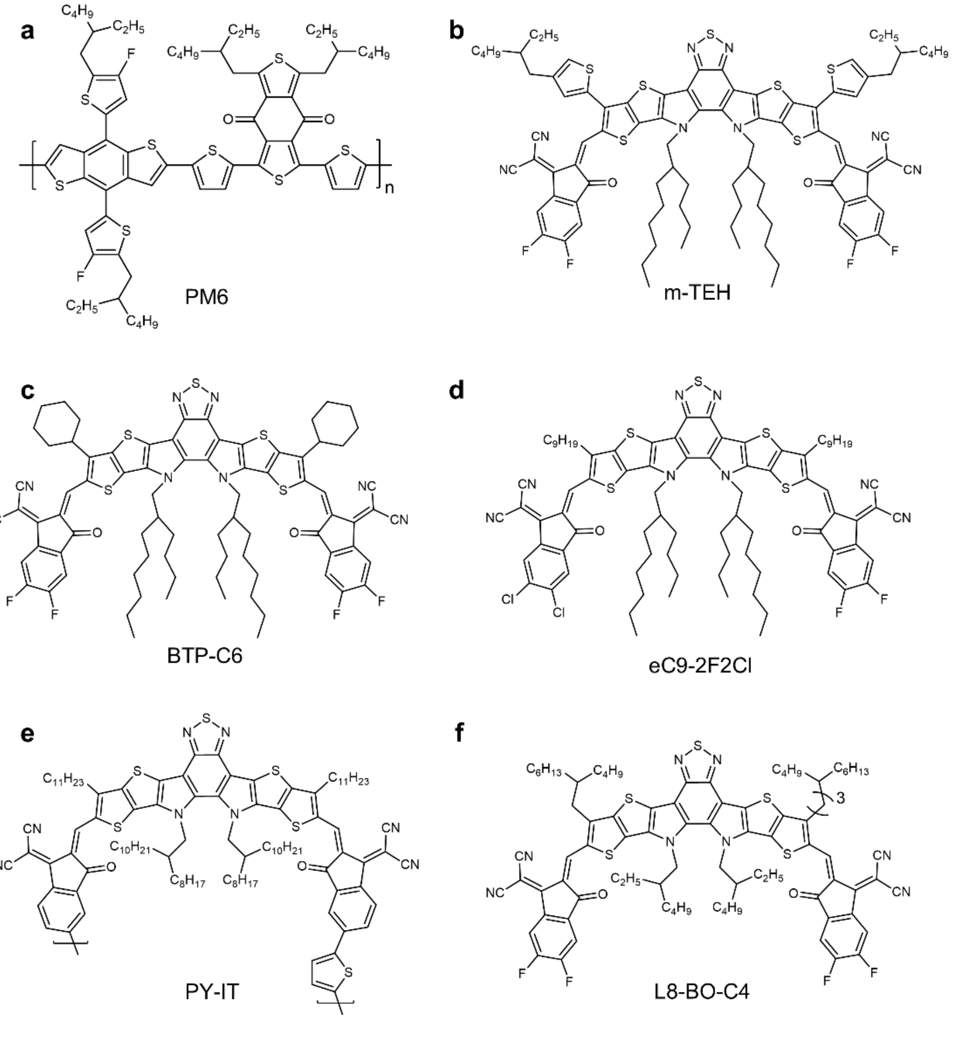

Supplementary Fig. 29. Chemical structures of the donor and acceptors.

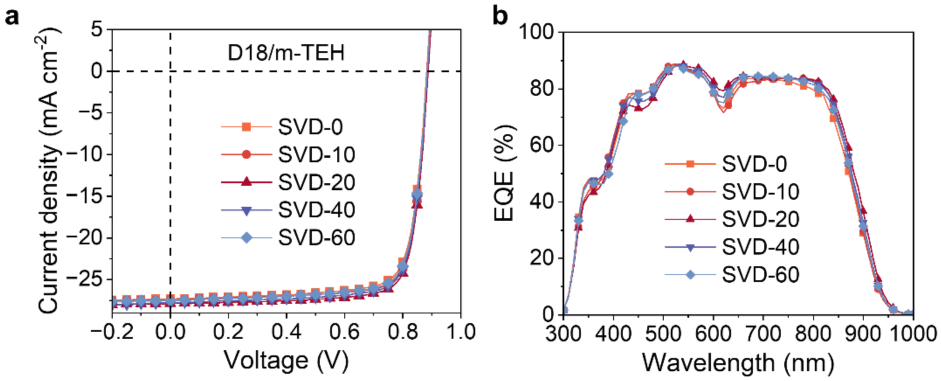

Supplementary Fig. 30. Photovoltaic performances of D18/m-TEH based OSCs. Chloroform was employed as the donor solvent, benzene/toluene was employed as the solvent pair for SVD treatment of m-TEH. **a** *J-V* curves. **b** EQE spectra.

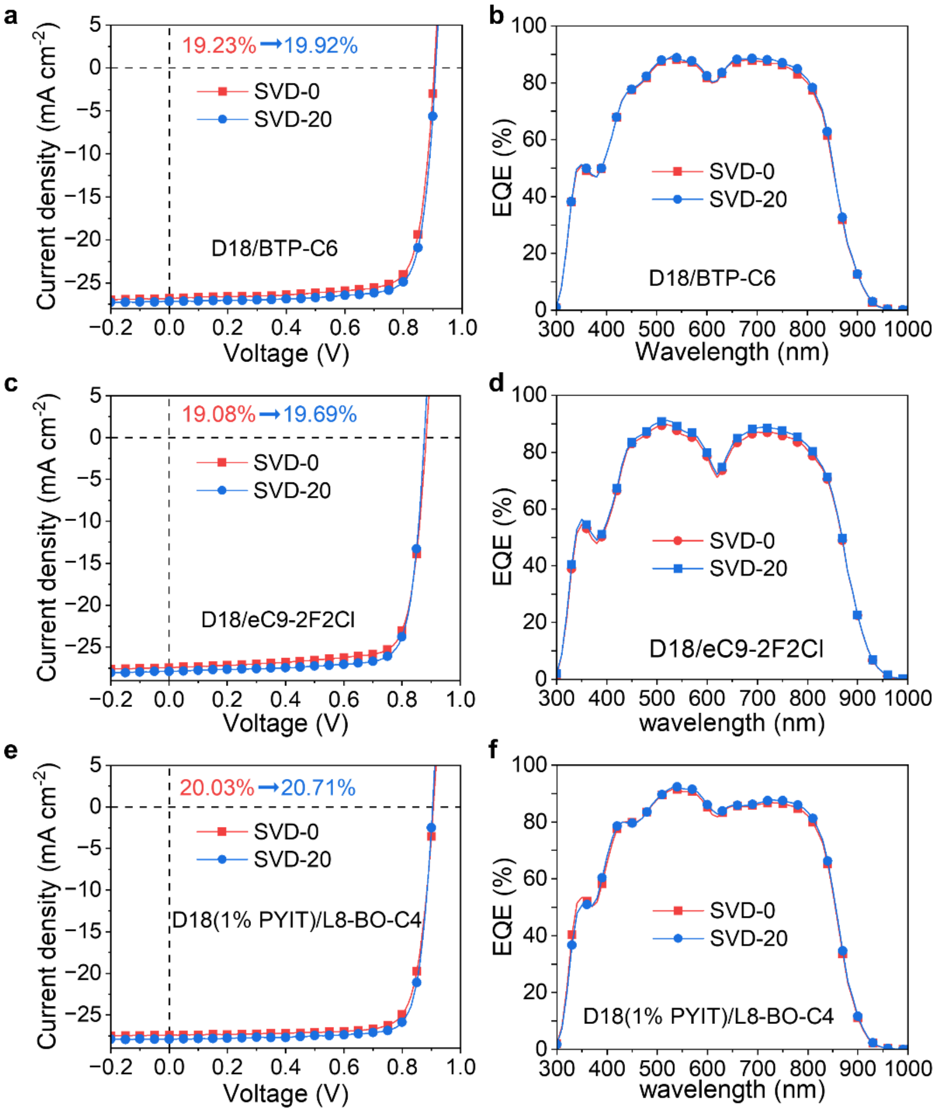

Supplementary Fig. 31. Photovoltaic performances of D18/NFA based OSCs. Chloroform was employed as the donor solvent, benzene/toluene was employed as the solvent pair for SVD treatment of NFAs. **a**,**c**,**e** *J*-*V* curves. **b**,**d**,**f** EQE spectra.

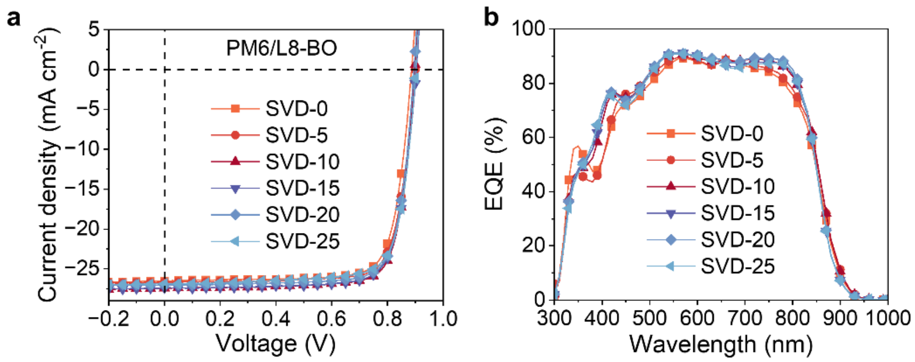

Supplementary Fig. 32. Photovoltaic performances of PM6/L8-BO based OSCs. Chlorobenzene was employed as the donor solvent, acetonitrile/toluene was employed as the solvent pair for SVD treatment of L8-BO. **a** *J-V* curves. **b** EQE spectra.

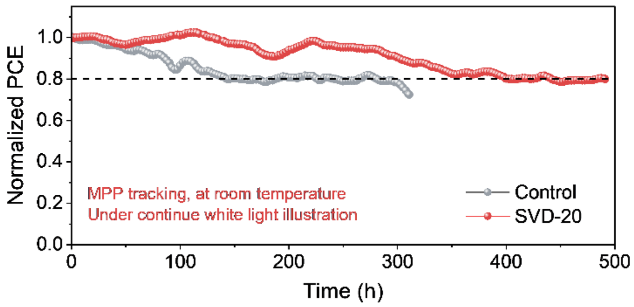

Supplementary Fig. 33. Maximum power point (MPP) tracking at room temperature under continuous white LED illumination (1 sun) for SVD-0 (control) and SVD-20 treated D18/L8-BO OSCs.

Supplementary Tables

Supplementary Table 1. Gas chromatography analysis of relative peak areas for pristine benzene, toluene, and toluene samples subjected to SVD in benzene atmosphere for different durations. Peak 1 corresponds to benzene and Peak 2 to toluene.

SVD time	Peak number	Relative peak area
Toluene	1	\
	2	100%
20 min	1	18.84%
	2	81.16%
40 min	1	32.21%
	2	67.79%
60 min	1	40.41%
	2	59.31%
120 min	1	55.76%
	2	44.24%
Benzene	1	100%
	2	\

Supplementary Table 2. UV–vis–NIR absorption spectra of L8-BO films deposited from solutions subjected to SVD for different durations.

SVD condition	Location		Intensity		$I_{0\text{-}0}/I_{0\text{-}1}$
	$Peak_{0\text{-}0}$ [nm]	$Peak_{0\text{-}1}$ [nm]	$Peak_{0\text{-}0}$ [$\times 10^5$ cm^{-1}]	$Peak_{0\text{-}1}$ [$\times 10^5$ cm^{-1}]	
SVD-0	805.2	714.4	1.20	0.81	1.48
SVD-10	805.6	715.0	1.22	0.81	1.50
SVD-20	806.4	716.3	1.24	0.81	1.54
SVD-40	807.7	720.1	1.27	0.78	1.62
SVD-60	808.5	719.4	1.29	0.79	1.64

Supplementary Table 3. Photovoltaic parameters of additive-free D18/L8-BO OSCs fabricated with L8-BO treated by SVD for different durations.

Active layer (without additive)	V_{oc}[a] [V]	J_{sc}[a] [mA cm^{-2}]	J_{EQE}[b] [mA cm^{-2}]	FF[a] [%]	PCE[a] [%]
D18/L8-BO	0.914	25.68	24.95	77.70	18.24
(SVD-0)	0.912±0.003	25.78±0.33		77.14±0.81	18.14±0.11
D18/L8-BO	0.910	26.27	25.38	78.24	18.71
(SVD-10)	0.911±0.005	26.17±0.24		78.15±0.34	18.63±0.07
D18/L8-BO	0.907	26.87	25.54	79.60	19.39
(SVD-20)	0.910±0.003	26.70±0.11		79.67±0.28	19.36±0.09
D18/L8-BO	0.911	26.44	25.26	79.10	19.04
(SVD-40)	0.912±0.003	26.29±0.15		79.04±0.29	18.95±0.08
D18/L8-BO	0.908	26.25	25.05	77.49	18.47
(SVD-60)	0.912±0.004	25.99±0.20		77.57±0.28	18.39±0.05
D18/L8-BO	0.918	24.61	24.11	75.82	17.13
(SVD-120)	0.917±0.005	24.46±0.37		75.42±0.95	16.91±0.22

aThe averaged values with standard deviations are calculated from ten individual devices. [b]Short current density values are calculated from EQE integration.

Supplementary Table 4. Photovoltaic performance of D18/L8-BO devices at Mix-0 and Mix-20 conditions.

Processing condition	V_{oc}[a] [V]	J_{sc}[a] [mA cm^{-2}]	J_{EQE}[b] [mA cm^{-2}]	FF[a] [%]	PCE[a] [%]
Mix-0	0.905	26.46	25.77	80.27	19.23
	0.902±0.002	26.57±0.79		80.09±0.20	19.19±0.06
Mix-20	0.902	26.89	25.78	79.73	19.33
	0.901±0.002	26.80±0.12		79.79±0.35	19.27±0.06

[a]The averaged values with standard deviations are calculated from ten individual devices. [b]Short-circuit current density values are calculated from EQE integration.

Supplementary Table 5. Ordered molecular structure parameters of the neat D18 film and D18/L8-BO films prepared with L8-BO treated by SVD for different durations.

Film	Lamellar stacking			π-π stacking		
	q/*d* [$Å^{-1}$]/[Å]	FWHM [$Å^{-1}$]	CCL [Å]	*q* [$Å^{-1}$]/[Å]	FWHM [$Å^{-1}$]	CCL [Å]
D18	0.291/21.59	0.057	148.1	1.58/3.98	0.507	16.65
SVD-0	0.313/20.07	0.081	104.2	1.67/3.77	0.236	35.76
SVD-20	0.314/20.01	0.062	135.2	1.67/3.76	0.225	37.51
SVD-40	0.314/20.01	0.059	142.5	1.66/3.79	0.219	38.54
SVD-60	0.312/20.14	0.058	145.5	1.66/3.79	0.211	39.99
SVD-120	0.315/19.95	0.054	156.3	1.65/3.80	0.198	42.62

Supplementary Table 6. Ordered molecular structure parameters of the D18/L8-BO films processed with L8-BO subjected to SVD or Mix treatments.

Film	Lamellar stacking			π-π stacking		
	q/*d* [$Å^{-1}$]/[Å]	FWHM [$Å^{-1}$]	CCL [Å]	*q*/*d* [$Å^{-1}$]/[Å]	FWHM [$Å^{-1}$]	CCL [Å]
SVD-0	0.316/19.88	0.078	108.2	1.68/3.75	0.236	35.71
SVD-20	0.314/20.01	0.061	138.3	1.66/3.78	0.190	44.45
SVD-120	0.317/19.82	0.053	158.3	1.67/3.77	0.167	50.70
Mix-0	0.316/19.88	0.063	133.9	1.68/3.74	0.208	40.65
Mix-20	0.314/20.01	0.063	134.8	1.67/3.77	0.199	42.21

Supplementary Table 7. Fitting parameters of 1D GISAXS profiles for D18/L8-BO films prepared with L8-BO subjected to SVD or Mix treatments.

D18/L8-BO	ξ [nm]	η [nm]	D	$2R_g$ [nm]
SVD-0	22.54	4.19	3.4	22.92
SVD-20	23.28	7.87	3.4	43.05
SVD-120	25.89	14.52	2.7	64.90
Mix-0	19.66	7.22	2.7	32.27
Mix-20	24.63	8.47	2.7	37.86

Supplementary Table 8. GTSAXS structural parameters at different grazing incidence angles for D18/L8-BO films prepared with L8-BO subjected to SVD or Mix treatments.

Processing condition	0.08°		0.12°		0.16°		0.2°	
	2Rg [nm]	φ [nm]	2Rg [nm]	φ [nm]	2Rg [nm]	φ [nm]	2Rg [nm]	φ [nm]
SVD-0	29.2	38.0	28.4	39.3	30.2	37.6	30.7	38.6
SVD-20	43.0	30.5	34.2	32.3	28.4	35.8	26.8	38.2
SVD-120	49.8	34.1	44.0	38.3	38.2	38.8	36.8	43.2
Mix-20	34.2	43.3	30.0	43.6	32.4	44.6	31.6	42.3

Supplementary Table 9. Charge carrier mobilities of D18/L8-BO films prepared with L8-BO subjected to SVD or Mix treatments.

Processing condition	Electron mobility[a] μ_e [cm^2 V^{-1} s^{-1}]	Hole mobility[a] μ_h [cm^2 V^{-1} s^{-1}]	Ratio μ_h/μ_e
SVD-0	3.50×10^{-4}	5.54×10^{-4}	1.556
SVD-20	9.04×10^{-4}	8.13×10^{-4}	0.899
SVD-120	2.19×10^{-4}	3.22×10^{-4}	1.467
Mix-0	7.22×10^{-4}	3.39×10^{-4}	0.544
Mix-20	7.31×10^{-4}	6.51×10^{-4}	0.890

[a]All devices were fabricated with thickness of 120 nm. The hole and electron mobilities were calculated as follows: $J=9\varepsilon_0\varepsilon_r\mu V^2/8L^3$, J is the current density, ε_r is the relative dielectric constant of the active layer, ε_0 is the permittivity of free space (8.85×10^{-14} F/cm), μ is the charge mobility, and L is the thickness of the active layer. V is the applied voltage. The ε_r parameter is assumed to be 3, which is a typical value for organic materials.

Supplementary Table 10. Photovoltaic parameters of D18/L8-BO based OSCs fabricated with L8-BO subjected to SVD for various durations (chloroform was employed as the donor solvent, dioxane/chlorobenzene was employed as the solvent pair for SVD treatment of L8-BO).

Active layer	V_{oc}[a] [V]	J_{sc}[a] [mA cm^{-2}]	J_{EQE}[b] [mA cm^{-2}]	FF[a] [%]	PCE[a] [%]
D18/L8-BO (SVD-0)	0.905	26.33	24.90	77.09	18.36
	0.906±0.006	25.89±0.35		77.62±0.48	18.19±0.12
D18/L8-BO (SVD-15)	0.910	26.38	25.15	77.91	18.71
	0.909±0.007	26.20±0.19		78.28±0.40	18.63±0.14
D18/L8-BO (SVD-30)	0.911	26.92	26.13	79.31	19.46
	0.910±0.005	26.65±0.26		79.57±0.51	19.30±0.10
D18/L8-BO (SVD-45)	0.911	26.43	25.22	79.06	19.02
	0.886±0.002	27.65±0.17		79.19±0.33	19.41±0.04
D18/L8-BO (SVD-60)	0.912	25.90	25.11	78.83	18.61
	0.905±0.011	26.23±0.32		77.85±0.87	18.47±0.19

[a]The averaged values with standard deviations are calculated from ten individual devices. [b]Short current density values are calculated from EQE integration.

Supplementary Table 11. Photovoltaic parameters of D18/m-TEH based OSCs fabricated with m-TEH subjected to SVD for various durations (chloroform was employed as the donor solvent, benzene/toluene was employed as the solvent pair for SVD treatment of m-TEH).

Active layer	V_{oc}[a] [V]	J_{sc}[a] [mA cm^{-2}]	J_{EQE}[b] [mA cm^{-2}]	FF[a] [%]	PCE[a] [%]
D18/m-TEH (SVD-0)	0.883	27.30	26.05	78.58	18.94
	0.883±0.002	27.26±0.14		78.42±0.30	18.88±0.17
D18/m-TEH (SVD-10)	0.887	27.48	26.27	79.53	19.38
	0.887±0.003	27.48±0.11		79.20±0.29	19.30±0.07
D18/m-TEH (SVD-20)	0.887	27.88	26.74	79.91	19.76
	0.886±0.002	27.76±0.09		79.54±0.36	19.56±0.11
D18/m-TEH (SVD-40)	0.885	27.78	26.59	79.25	19.49
	0.886±0.002	27.65±0.17		79.19±0.33	19.41±0.04
D18/m-TEH (SVD-60)	0.884	27.50	26.29	78.86	19.18
	0.886±0.003	27.37±0.18		78.70±0.37	19.08±0.08

[a]The averaged values with standard deviations are calculated from ten individual devices. [b]Short current density values are calculated from EQE integration.

Supplementary Table 12. Photovoltaic performance of OSCs fabricated using NFAs subjected to SVD and non-SVD treatments.

Active layer	V_{oc} [a] [V]	J_{sc} [a] [mA cm^{-2}]	J_{EQE} [b] [mA cm^{-2}]	FF [a] [%]	PCE [a] [%]
D18/BTP-C6	0.905	26.80	25.71	79.28	19.23
	0.907±0.002	26.51±0.29		79.71±0.28	19.16±0.20
D18/BTP-C6 (SVD)	0.910	27.16	25.94	80.62	19.92
	0.910±0.001	26.97±0.20		80.56±0.10	19.78±0.14
D18/eC9-2F2Cl	0.883	27.44	26.24	78.73	19.08
	0.878±0.007	27.39±0.18		78.00±0.48	18.99±0.13
D18/eC9-2F2Cl (SVD)	0.877	27.90	26.67	80.48	19.69
	0.873±0.003	27.88±0.14		80.36±0.41	19.57±0.08
D18 (1% PYIT)/ L8-BO-C4 [c]	0.907	27.43	26.28	80.52	20.03
	0.905±0.003	27.17±0.22		80.54±0.38	19.80±0.16
D18 (1% PYIT)/ L8-BO-C4 (SVD) [c]	0.904	27.90	26.53	82.12	20.71
	0.903±0.002	27.83±0.07		81.94±0.28	20.60±0.08

[a]The averaged values with standard deviations are calculated from ten individual devices. [b]Short current density values are calculated from EQE integration. [c]1% PYIT was added to the donor D18 solution during the device preparation process.

Supplementary Table 13. Photovoltaic parameters of PM6/L8-BO based OSCs fabricated with L8-BO subjected to SVD for various durations (chlorobenzene was employed as the donor solvent, acetonitrile/toluene was employed as the solvent pair for SVD treatment of L8-BO).

Active layer	V_{oc}[a] [V]	J_{sc}[a] [mA cm^{-2}]	J_{EQE}[b] [mA cm^{-2}]	FF[a] [%]	PCE[a] [%]
PM6/L8-BO (SVD-0)	0.887	26.52	25.21	78.74	18.52
	0.886±0.003	26.35±0.18		79.02±0.33	18.45±0.07
PM6/L8-BO (SVD-5)	0.900	26.70	25.64	78.84	18.94
	0.889±0.007	26.74±0.12		78.73±0.28	18.72±0.16
PM6/L8-BO (SVD-10)	0.898	27.44	26.31	79.40	19.58
	0.896±0.004	27.34±0.14		79.28±0.33	19.43±0.10
PM6/L8-BO (SVD-15)	0.904	27.41	26.29	78.27	19.39
	0.893±0.004	27.12±0.19		78.95±0.54	19.12±0.15
PM6/L8-BO (SVD-20)	0.895	27.03	26.04	79.21	19.17
	0.893±0.003	26.96±0.16		78.98±0.56	19.02±0.14
PM6/L8-BO (SVD-25)	0.902	26.88	25.92	77.98	18.90
	0.902±0.002	26.73±0.18		77.49±0.58	18.68±0.20

[a]The averaged values with standard deviations are calculated from ten individual devices. [b]Short current density values are calculated from EQE integration.
