# Supplementary material for: Solvent vapor diffusion–driven multiscale pre-aggregation of non-fullerene acceptors enables high-performance organic solar cells
Source: Nat Commun. 2025 Dec 17;16:11188. doi: 10.1038/s41467-025-66199-5 (PMC12712035; doi:10.1038/s41467-025-66199-5)
Supplement: Supplementary file 2 — Reporting Summary [file 41467_2025_66199_MOESM2_ESM.pdf]

nature portfolio

Corresponding author(s): Qiang Peng

Solar Cells Reporting Summary

Nature Portfolio wishes to improve the reproducibility of the work that we publish. This form is intended for publication with all accepted papers reporting the characterization of photovoltaic devices and provides structure for consistency and transparency in reporting. Some list items might not apply to an individual manuscript, but all fields must be completed for clarity.

For further information on Nature Research policies, including our data availability policy, see Authors & Referees.

Experimental design

Please check the following details are reported in the manuscript, and provide a brief description or explanation where applicable.

1. Dimensions

Area of the tested solar cells

- [x] Yes — Aperture area of 0.04 cm2 was used in this work.
- [ ] No — *Explain why this information is not reported/not relevant.*

Method used to determine the device area

- [x] Yes — The area of devices was determined by a mask.
- [ ] No — *Explain why this information is not reported/not relevant.*

2. Current-voltage characterization

Current density-voltage (J-V) plots in both forward and backward direction

- [ ] Yes
- [x] No — The hysteresis effect of organic solar cells can be ignored, and the positive and negative scanning results are the same.

Voltage scan conditions

- [x] Yes — The condition of voltage scanning is forward scanning at a speed of 0.01 V and dwell time of 1 ms.
- [ ] No — *Explain why this information is not reported/not relevant.*

Test environment

- [x] Yes — The devices were characterized at room temperature in a glove box.
- [ ] No — *Explain why this information is not reported/not relevant.*

Protocol for preconditioning of the device before its characterization

- [ ] Yes — *Provide a description of the protocol.*
- [x] No — No preconditioning was used in this work.

Stability of the J-V characteristic

- [x] Yes — Stabilized PCE of the devices were provided Supplementary Fig. 33.
- [ ] No — *Explain why this information is not reported/not relevant.*

3. Hysteresis or any other unusual behaviour

Description of the unusual behaviour observed during the characterization

- [ ] Yes — *Provide a description of hysteresis or any other unusual behaviour observed during the characterization.*
- [x] No — No any the unusual behaviour observed during the characterization.

Related experimental data

- [ ] Yes — *Provide a description of the related experimental data.*
- [x] No — No any the unusual behaviour observed during the characterization.

4. Efficiency

External quantum efficiency (EQE) or incident photons to current efficiency (IPCE)

- [x] Yes — This information can be found in manuscript and Supplementary Information.
- [ ] No — *Explain why this information is not reported/not relevant.*

A comparison between the integrated response under the standard reference spectrum and the response measure under the simulator

- [x] Yes — The integrated Jsc values were consistent with Jsc values from the J-V curves.
- [ ] No — *Explain why this information is not reported/not relevant.*

For tandem solar cells, the bias illumination and bias voltage used for each subcell	☐ Yes<br>☒ No	*Provide a description of the measurement conditions.*<br>Tandem solar cells were not covered in this work.

5. Calibration

Light source and reference cell or sensor used for the characterization	☒ Yes<br>☐ No	See methods.<br>*Explain why this information is not reported/not relevant.*
Confirmation that the reference cell was calibrated and certified	☒ Yes<br>☐ No	The standard silicon solar cell calibrated by Newport was used to calibrate light intensity to AM 1.5G.<br>*Explain why this information is not reported/not relevant.*
Calculation of spectral mismatch between the reference cell and the devices under test	☐ Yes<br>☒ No	*Provide a value of the spectral mismatch and/or a description of how it has been taken into account in the measurements.*<br>*Explain why this information is not reported/not relevant.*

6. Mask/aperture

Size of the mask/aperture used during testing	☒ Yes<br>☐ No	Metal mask with area of 0.04 cm2 was used.<br>*Explain why this information is not reported/not relevant.*
Variation of the measured short-circuit current density with the mask/aperture area	☐ Yes<br>☒ No	*Report the difference in the short-circuit current density values measured with the mask and aperture area.*<br>We measure all devices with metal mask.

7. Performance certification

Identity of the independent certification laboratory that confirmed the photovoltaic performance	☒ Yes<br>☒ No	Certification was provided by National Photovoltaic Product Quality Insoection&Testing Center, China.<br>*Explain why this information is not reported/not relevant.*
A copy of any certificate(s)	☐ Yes<br>☒ No	*Certificate copies should be provided in the Supplementary information. Please state the supplementary item number.*<br>*Explain why this information is not reported/not relevant.*

8. Statistics

Number of solar cells tested	☒ Yes<br>☐ No	10 devices. This information can be found in manuscript and Supplementary Information.<br>*Explain why this information is not reported/not relevant.*
Statistical analysis of the device performance	☒ Yes<br>☐ No	The average PCE were calculated from 10 devices for each OSC. This information can be found in manuscript and Supplementary Information.<br>*Explain why this information is not reported/not relevant.*

9. Long-term stability analysis

Type of analysis, bias conditions and environmental conditions	☒ Yes<br>☐ No	See methods.<br>*Explain why this information is not reported/not relevant.*
